# Investing in early years to reduce non-communicable diseases in adults

**DOI:** 10.1093/eurpub/ckab203

**Published:** 2021-11-24

**Authors:** Ruth Dundas, Alastair H Leyland

**Affiliations:** MRC/CSO Social and Public Health Sciences Unit, University of Glasgow, Glasgow, UK

Health and wellbeing during the early years of life have a crucial impact on chronic disease (such as cardiovascular, mental health and obesity) and inequalities, across the lifecourse and from one generation to the next. There is clear evidence of the impact of intrauterine and infant growth, and pre-term birth on adult cardiovascular health.^1^ Investing early and throughout childhood provides economic benefits including improved health and reduced spending for society.^2^ WHO Europe’s Investing in Children strategy recognizes the importance of a lifecourse approach to improving adult health and many governments’ strategies and policies are aimed at the early years of life.^3^ To ensure these investments improve health and reduce inequalities, and to enable other countries to learn from successes, these policies need to be evaluated.

Investing in early years to realize improvements in adult health could mean waiting for years to discover the full impact of current policies or relying on evaluations of the impact of older policies on current adult health. Policies becoming outdated may limit the utility of evidence from the latter. Focusing on the former does not necessitate waiting before undertaking evaluations of such policies. They can be evaluated using intermediate health outcomes early in the lifecourse because the evidence for the relationship between child health and adult health is well established.

Robust evaluations of current policies that affect health outcomes early in the lifecourse should be undertaken to ensure any investment is being spent on the right policy. The preferred option from the viewpoint of evaluation is to have a randomized rollout of a policy. However, this is rarely feasible, ethical or practical. In place of randomization, we can use natural experimental methods which offer the opportunity to evaluate policies that vary between regions and countries. The introduction of a policy in a single country can also be evaluated, but the design—an uncontrolled before and after study—is weaker. Such evaluations require (i) assessment of the policy context/landscape; (ii) identification of outcomes and development of logic models; (iii) use of appropriate design and evaluation methods; (iv) identification of exposed and comparator groups; (v) identification of data sources; and (vi) conduct of an economic evaluation alongside outcome evaluation.

Different levels of government within and between countries may be responsible for the introduction and implementation of policies. Policies may interact with each other and isolating the effect of a single policy may be difficult. We need to recognize the wider landscape to understand what other policies are in place at different times (within a country) or in different contexts (between countries). The determinants of infant and child health are complex and inter-related, ranging from family socio-economic circumstances and childcare settings to neighbourhood deprivation and pollution, therefore many policies that may affect the early years (and adult NCDs) will be from sectors other than health, such as welfare, employment, housing, education and physical environment.^4^

In order to conduct robust evaluations, we must be able to identify the exposed and a comparable unexposed group. This can be difficult at the population level, when outcome data, such as health records, are not linked to exposure data (e.g. welfare policies). Instead, eligibility criteria can be used to construct an exposed and an unexposed control group. The exposed group does not need to comprise those who actually receive the policy, but only those who meet eligibility criteria. For example, policies aimed at supporting all teenage mothers need only be able to identify teenage mothers and not only those teenage mothers who took up the intervention. This is can be thought of as an intention to treat analysis, demonstrating how the policy works in practice. Administrative records, where they exist, covering the social determinants from different sectors, covering families and the lifecourse, are an ideal data source. No primary data collection is required meaning that any policy can be evaluated, not only those which require planning to collect data prior to policy implementation. This requires governments to make administrative data linked across sectors available to researchers.

In addition to outcome evaluations, economic evaluations should also be conducted.^5^ There are convincing arguments that investment earlier in the lifecourse provides more value for societal benefits. This can be empirically tested in economic evaluations and will be useful for future policy investment.

Infant and child health is important for adult health, and there is a willingness across governments to spend in this area, but robust outcome evaluations are needed to ensure money is spent on policies that will improve health and reduce health inequalities.
